# Multi-Color Printed Textiles for Ultraviolet Radiation Measurements, Creative Designing, and Stimuli-Sensitive Garments

**DOI:** 10.3390/ma16165622

**Published:** 2023-08-14

**Authors:** Elżbieta Sąsiadek-Andrzejczak, Marek Kozicki

**Affiliations:** Department of Mechanical Engineering, Informatics and Chemistry of Polymer Materials, Lodz University of Technology, Żeromskiego 116, 90-543 Lodz, Poland

**Keywords:** textile printing, three-color printing, UV dosimeters, UV radiation monitoring, stimuli-sensitive garments, creative designing of textiles

## Abstract

This work concerns the new idea of textile printing with a multi-color system using pastes containing compounds sensitive to ultraviolet (UV) radiation. A screen printing method based on a modified CMYK color system was applied to a cotton woven fabric. Aqueous printing pastes were prepared from thickening and crosslinking agents and UV-sensitive compounds: leuco crystal violet (LCV), leuco malachite green (LMG), and 2,3,5-triphenyltetrazolium chloride (TTC) instead of the system’s standard process colors: cyan, magenta, and yellow. Depending on the number of printed layers and the type of UV radiation (UVA, UVB, and UVC), the modified textile samples change color after irradiation from white to a wide range of colors (from blue, red, and green to purple, brown, and gray). Based on reflectance measurements, the characteristic parameters of the one-, two-, and three-color-printed samples in relation to absorbed dose were determined, e.g., dose sensitivity, linear and dynamic dose response, and threshold dose. This printing method is a new proposal for UV dosimeters and an alternative standard for textile printing. Furthermore, the developed method can be used for the securing, marking, and creative design of textiles and opens up new possibilities for such stimulus-sensitive reactive printing.

## 1. Introduction

Textile printing is a well-known method of finishing textiles for various purposes, including: clothing, workwear, and decorative textiles such as wallpapers, upholstery materials, curtains, tablecloths, etc. There are different methods of textile printing, e.g., manual and mechanical. The print is usually transferred to the selected textile substrate by (i) patterned wooden, metal, or rubber stamps; (ii) flat or rotary screens made of wood, metal or plastic; (iii) pattern spray nozzles called digital printing. These methods are described in detail in the literature and are also used in the production of paper, films, ceramics, and plastics [1,2,3,4]. The screen printing process is simple, cheap, and also suitable for printing sensors and markers that can be used to identify and secure various textile products against uncontrolled copying or counterfeiting.

Production sectors such as food, cosmetics, or electronics treat product safety as one of the basic quality characteristics that depends on factors such as the quality of the raw material for production, the correctness of the production process, and the established storage, transport, and distribution conditions. They use some indicators that provide information about changes in humidity, temperature, oxygen reduction, overexposure to the sun’s radiation (including UV radiation), and many other factors that affect the quality of the products. At the same time, they ensure the proper functioning of supply chains, from the sourcing of individual ingredients to the final product [5]. However, these are not the indicators associated with protection of the products from unauthorized copying. For instance, high-value products such as money and securities are often marked with (i) luminescent; (ii) infrared radiation absorbing; (iii) photochromic; (iv) thermochromic; (v) penetrating; (vi) optically variable; (vii) iridescent; or (viii) chemically reactive paints. However, no similar solutions on securing textiles was found in the literature.

Among the printed markers are also UV indicators, which are used to monitor ultraviolet (UV) radiation. In general, they provide information on radiation dose in a simple, visible, and quick way without the need to use special measuring equipment. The indicators are based on the use of radiation-sensitive compounds that change color after UV irradiation and can be compared with a colored pattern. However, such markers do not provide information about the radiation dose [6]. Recently, our research group proposed dosimeters based on tetrazolium salts and diacetylenes to measure the 2D and 3D dose distribution of UV radiation. The original solution for UV radiation-sensitive textile dosimeters based on a single color printing process with 2,3,5-triphenyltetrazolium chloride (TTC) and blue color forming nitrotetrazolium blue chloride (NBT) was also published [7]. It has been shown that cotton fabric printed with a paste containing a single colored promoter changes its color from colorless to red/orange (printing paste with TTC), and from colorless to violet/blue (printing paste with NBT) as a result of irradiation. The intensity of the color depends on the absorbed UV dose. The higher the absorbed dose or the concentration of color promotors, the more intense the color of the samples. It has also been shown that cotton fabric samples printed with TTC paste can be used to monitor UV doses up to 0.5 J/cm^2^, while polyamide fabric samples printed with NBT can be used up to 3 J/cm^2^. In addition, two color precursors can be combined to obtain a wider measurement range for UV radiation doses up to 10 J/cm^2^. Furthermore, the results showed that the developed systems can be used to obtain a realistic exposure level for UV radiation measurements, including the factors SED (Standard Erythema Dose) and MED (Minimum Erythema Dose). For example, the factors SED and MED are 10 mJ/cm^2^ and 25 mJ/cm^2^, respectively, in Barcelona (Spain) [8], which are within the linear range of measured UV doses analyzed in our previous research. Although the possibilities of single- and two-color printing have been shown in previous works, the subject has not yet been fully exhausted.

The approach in this work was to modify the surface of textiles using the concept of a four-color CMYK printing technique to obtain a multi-color UV sensor that can be used as a garment for people exposed to radiation and/or as a decorative element. CMYK color printing is a well-known technique used in the polygraphy industry but has little importance in the production of textiles. Therefore, it was interesting to develop a dosimeter based on a system similar to the CMYK subtractive color mixing system (a printing system with cyan, magenta, yellow, and black), which changes its color after UV exposure in a wide range of doses. By using a system of three color precursors and black printing paste (which is not sensitive to radiation), it is possible to create a dosimeter with a wide range of sensitivity to radiation doses and to obtain a multi-color image through color mixing. The dosimeters that are developed in terms of their use as part of the product, including as: (i) a personal dosimeter for protection against UV radiation; (ii) a marker for the originality of products (finished products, e.g., outerwear, workwear, textile raw materials, and packaging); (iii) a marker for authenticity of textiles or other products; (iv) decorative, ornamental elements; (v) a product freshness indicator—element of food or cosmetic packaging; and (vi) a design detail element and originality marker, e.g., securities. Following previous research [7,9], cotton woven fabric was selected for modification by a screen-printing process. Instead of single-color printing, the CMYK four-color printing technique was used, and printing pastes containing a thickener, a binder, and three radiochromic color precursors were selected to mimic the use of the commercial colors: leuco crystal violet (LCV) instead of cyan, 2,3,5-triphenyltetrazolium chloride (TTC) instead of magenta, and leuco malachite green (LMG) instead of yellow. Only the black color was not changed because it was used as a contour color that should not be affected by UV radiation. Due to the chemical structure of leuco dyes and tetrazolium salts, it is not possible to produce such color promoters that, under the influence of UV radiation, give a color palette similar to the traditional CMYK color system [10]. It should also be emphasized that the color created by the reaction of LCV, TTC, and LMG with UV radiation does not replace the CMYK system. The proposed three radiochromic components of printing pastes allowed us to obtain color changes in the red/orange, blue, and green/yellow ranges (BRKG) and develop dosimeters with a wide measuring range for different types of UV radiation (UVA, UVB, and UVC). It is important that, in the presented printing method, LCV, TTC, and LMG are used only as example compounds. In further work, the use of other leuco dyes, including other tetrazolium salts, or the use of other groups of radiation-sensitive dyes or polydiacetylenes may be considered as alternatives to the currently used compounds in dyeing and printing methods. Reports of possible skin irritation from the leuco dyes used in the research should be taken into consideration. Studies on the toxicity of various leuco dyes after intravenous, subcutaneous, or intraperitoneal injection are discussed elsewhere [11]. Although a printed pattern is not usually in direct contact with the skin, skin irritation from LCV, TTC, and LMG should not be neglected. In addition, different printing pastes should be investigated, and changes in the components of the printing pastes should be considered. The change of color promoters or the addition of UV radiation retardants can lead to the production of systems with a wider measuring range. Further investigation of the durability of such prints may also provide clues about a new group of compounds to support UV color printing on textiles.

The aim of this work is to present the method of textile printing with the BRGK color system. At first, the cotton fabric samples were printed with single-, two-, and three-colors pastes containing radiation-sensitive color promoters. Afterwards, samples were irradiated with UV radiation and investigated with respect to the obtained calibration and basic characteristics of the dose response. Color changes due to irradiation were examined for different UV subranges and the number of printed layers on a textile substrate. The reflectance spectrophotometry was used to analyze the color of the developed dosimeters. Finally, several examples of applications related to personal protection against UV radiation, marking, labeling, securing, decoration, and design are also presented.

## 2. Materials and Methods

### 2.1. Preparation of Printing Paste

In this study, a white cotton fabric (twill weave; surface mass of 250 g/m^2^, thickness of 0.68 mm, weft setting 220/dm, and warp setting 240/dm) that was mercerized, sanforized, and bleached (Ten Cate, Almelo, The Netherlands) was used. The printing process was carried out with an aqueous printing paste consisting of Helizarin Binder (BASF, Ludwigshafen, Germany), Lutexal Hit (density at 20 C: approx. 1.0 g/cm^3^; BASF, Ludwigshafen, Germany), and three radiation-sensitive compounds: LCV (M = 373.53 g/mol; Sigma, Saint Louis, MI, USA); LMG (M = 330.47 g/mol; Sigma-Aldrich, Saint Louis, MI, USA); and TTC (M = 334.81 g/mol; Sigma-Aldrich, Saint Louis, MI, USA). Various additives were also used in the preparation of the LCV and LMG solutions: trichloroacetic acid (TCAA, Sigma-Aldrich, Saint Louis, MI, USA) at concentrations of 2.21% and 1.37%, and 0.16% of 4-(1,1,3,3-tetramethylbutyl) phenyl-polyethylene glycol (Triton X-100, Sigma-Aldrich, Saint Louis, MI, USA). The printing paste was prepared as follows. A mixture of 100 g Helizarin Binder and 32 g Lutexal Hit was mixed with 827 g distilled water. After 20 min of continuous mixing at a rotation speed of 1000 rpm, the stock paste was homogeneous and ready for use. The radiation-sensitive components were added to 300 g of the paste. The concentration of LCV, TTC, and LMG was the same in all printing pastes and amounted to 0.1%. The concentration of all components made it possible to obtain pastes that formed a transparent layer on the textile surface without visible initial color (all printed samples were white after printing before UV irradiation).

### 2.2. Printing

In this study, the screen printing method was used. This method can be used to print on various materials such as textiles, paper, plastics, glass, ceramics, and metals. All the stencils used (EX 63-063/160 PW screen: 63 mesh/cm; NBC, Tokyo, Japan; distributed by K+L Company, Lodz, Poland) were prepared with the aid of a photopolymerizable emulsion (Fotocoat 1010; Forteco, Kwintsheul, The Netherlands) on a woven polyester mesh stretched over an aluminum frame. The screen was coated with the photopolymerizable emulsion and dried in a dark cabinet at 30 °C (24 h). After drying, the screen was ready to be exposed to light and create a pattern. The designed pattern, simple rectangles (40 mm × 60 mm), and CMYK images of birds, were created as digital graphic files and printed on transparent film. All patterns were opaque (black color). In the case of the CMYK bird image, color separation was completed, and the resulting patterns were printed separately on the screens. Then, the films with the patterns were placed on the prepared screens and exposed to a light source (20 min; halogen lamp, Halogenfluter 500 W 930037; Düwi GmbH, Breckerfeld, Germany). After exposure, the screen was washed and dried. The cotton fabric samples were printed with a printing paste prepared as described in Section 2.1. Due to differences in the preparation of solutions of radiation-sensitive compounds, three pastes were used for multi-color prints to obtain a wide range of color changes after UV irradiation: blue from LCV, red from TTC, and green from LMG. To obtain different color changes, the cotton fabric was printed 1, 2, or 3 times, creating color combinations (1 layer: blue; green; red; 2 layers: blue + green; blue + red; green + blue; 3 layers: blue + green + red). In the case of the CMYK bird image, the color scheme was the same, but in addition, black non-radiation-sensitive paste was used. Thus, a color system based on blue, red, green, and black was created and called the BRGK system. The printing pastes were forced to move over a stencil using a medium-soft squeegee. The printed samples were then dried in a dryer (E. Benz, Stuttgart, Germany) at 30 °C for 60 min. The pick-up of the paste using a 1 g cotton sample was equal to 0.3990 g, 0.7511 g, and 1.0984 g for one-, two-, and three-color-printed samples, respectively, (rough estimation; no special conditioning of samples was applied). The prepared samples were ready for irradiation. After drying, the samples were covered in aluminum foil and protected from light. The packaging protected printed samples from accidental irradiation.

### 2.3. Irradiation

All printed cotton fabric samples were irradiated in the UV curing cabinets (UVP, Upland, CA, USA) at three wavelengths: UVA (8 W, type F8T5 Blacklight, range 315–400 nm; a peak at 369 nm, Hitachi, Tokyo, Japan); UVB (8 W, type G8T5E, range: 280–360 nm; a peak at 306 nm, Sankyo Denki, Tokyo, Japan); and UVC (8 W, type G8T5, 253.7 nm, Sankyo Denki, Tokyo, Japan). The samples were irradiated with UVA, UVB, and UVC in a dose range of 0–10 J/cm^2^. A given UV dose (J/cm^2^) was automatically delivered via a built-in detector and the control system of the device. For example, to emit a dose of 1 J/cm^2^, the time was 214 s, 190 s, and 330 s for UVA, UVB, and UVC radiation, respectively.

### 2.4. Measurements of Samples

The reflectance spectra of the cotton textile samples printed with LCV, LMG, and TTC pastes were measured with a Spectraflash light reflectance instrument (Spectraflash 300, D65⁄10; 10 nm resolution; the measurement error is 0.1%; DataColor, Rotkreuz, Switzerland). The device was calibrated beforehand, and the UV light of 190–400 nm was automatically cut off by the software (microMATCH v. 3.6; DataColor) after the 0% UV option was selected to avoid unnecessary irradiation of the samples. The samples were measured immediately after irradiation and over time after irradiation (for 30 days) over the wavelength range 400–700 nm. The wavelength at which the change in reflectance was maximum was then selected and discussed in relation to the absorbed UV dose. Based on the reflectance measurements, the characteristic parameters of the dosimeters were determined: dose response, dose sensitivity, linear and dynamic dose response, and threshold dose. In addition, the color coordinates were determined with the CIE Lab color system, which describes the perceived color according to the standard ISO/CIE 11664-4 [12].

### 2.5. Scanning Electron Microscopy Analysis

The morphology of unprinted and three-color-printed cotton samples both before and after UVB irradiation was analyzed using scanning electron microscopy (TESCAN VEGA3–EasyProbe, TESCAN Brno, s.r.o., Brno, Czech Republic) with a VEGATG software (high vacuum mode (SE); accelerating volt-age 20 kV). Before the measurements, the samples were coated with Au-Pd layers using a Cressington Sputter Coater 108 auto system (Cressington Scientific Instruments Ltd., Watford, UK).

## 3. Results and Discussion

### 3.1. One-Color Printing

**Figure 1:**
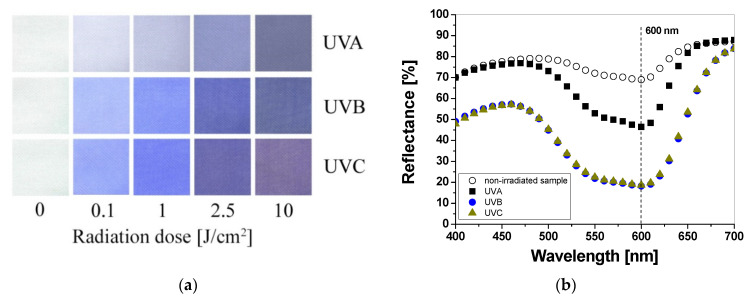
Photograph of cotton samples printed with LCV-containing printing paste and irradiated with UV light (UVA 369 nm; UVB 306 nm; and UVC 253.7 nm): 0, 0.1, 1, 2.5, and 10 J/cm^2^ (**a**) and reflectance spectra of the printed samples that were non-irradiated and irradiated with 1 J/cm^2^ UVA, UVB, and UVC radiation (**b**).

**Figure 2:**
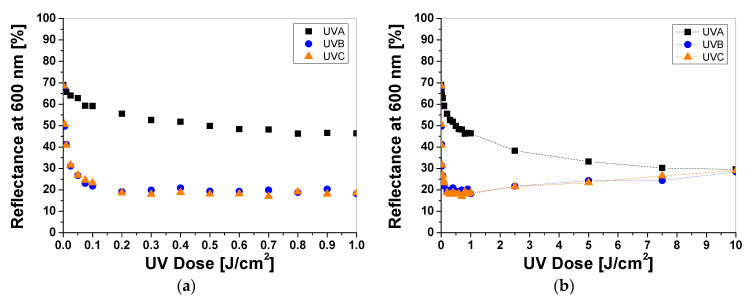
The reflectance of light at 600 nm vs. absorbed UV dose of printed cotton samples after UV irradiation (doses of UVA, UVB, and UVC in the range up to 1 J/cm^2^; samples were printed with LCV-containing paste and dried for 60 min at 30 °C) (**a**). Changes in light reflectance at 600 nm of samples irradiated with doses up to 10 J/cm^2^ of UV light (the increase in light reflectance is due to the color bleaching) (**b**).

Cotton samples printed with paste containing LCV and irradiated with UVA, UVB, and UVC change color from colorless to blue/purple. The intensity of the color depends on the type of UV radiation and the absorbed radiation dose. Regardless of the type of UV radiation, the color intensity of the sample increases with the radiation dose (Figure 1a). The reflectance of the light decreases with the absorbed dose in the wavelength range of 450–660 nm, with a maximum at 600 nm (Figure 1b). The color intensity is related to the absorbed UV dose. The higher the dose, the more intense the blue color (Figure 1a and Figure 2). It can also be seen that the color change of the samples is more visible in those that were irradiated with UVB and UVC radiation. The sensitivity of the dosimeter for UVA is the lowest of all UV subranges (Figure 2a,b).

For example, the difference in light reflectance between the samples irradiated with UVA and UVB at a dose of 0.1 J/cm^2^ equaled approx. 63%, while the difference between the UVB- and UVC-irradiated samples equaled approx. 6%. The intensity of the blue color reaches a plateau above 0.5 J/cm^2^ (Figure 2a). In the dose range of 0.5–1 J/cm^2^ no significant changes in reflectance was observed, indicating that saturation of the color occurred. It should be noted that irradiating the dosimeters with high doses of UVB and UVC radiation (over 1 J/cm^2^) increases the light reflectance of the samples, which could be related to bleaching (Figure 2b). The mechanism of the color change of the LCV from colorless to deep blue/purple can be attributed to the formation of the strongly colored quinoid chromophore as part of the resonant carbonium cation [13,14,15]. Similar observations have been described by the authors elsewhere for dosimetry systems for UV and ionizing radiation containing LCV [16], indicating the probable mechanism of these reactions. No bleaching effect was observed for the samples irradiated with UVA for the examined dose range. To describe the color differences of the individual samples in detail, the CIE Lab values of selected LCV printed samples irradiated with doses of 0, 0.1, 1, and 10 J/cm^2^ were compared and presented in Table 1. Comparing the CIE Lab with LCV-printed samples irradiated with higher doses of UVB and UVC, a change in color from blue to a more purple color is clearly visible.

**Figure 3:**
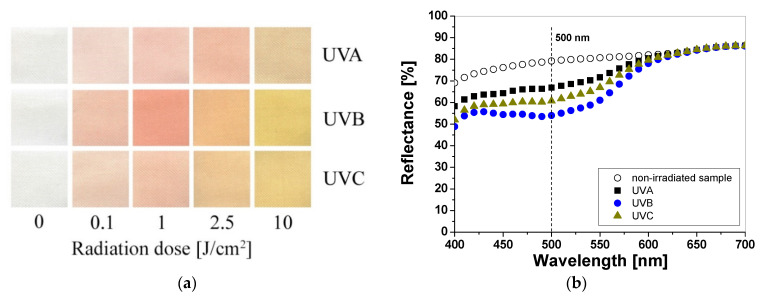
Photograph of cotton samples printed with TTC-containing printing paste and irradiated with UV light (UVA 369 nm; UVB 306 nm; and UVC 253.7 nm): 0, 0.1, 1, 2.5, and 10 J/cm^2^ (**a**) and reflectance spectra of the printed samples that were non-irradiated and irradiated with 1 J/cm^2^ of UVA, UVB, and UVC radiation (**b**).

Samples printed with paste containing TTC turn red/orange after UV irradiation, and the intensity of the color depends on the absorbed UV dose. The higher the absorbed dose, the more intense the color of the samples (Figure 3a). The color change is related to the transformation of the tetrazole ring of the TTC substrate through the reduction process and the formation of a corresponding formazan, which has been described elsewhere [17,18,19]. Similar to LCV, the cotton samples were measured to assess the influence of the absorbed UV dose on the light reflectance from the samples. The obtained results show that the change in the light reflectance for non-irradiated and irradiated samples changes over a wide range of wavelengths (420–620 nm) with a maximum at 500 nm (Figure 3b).

**Figure 4:**
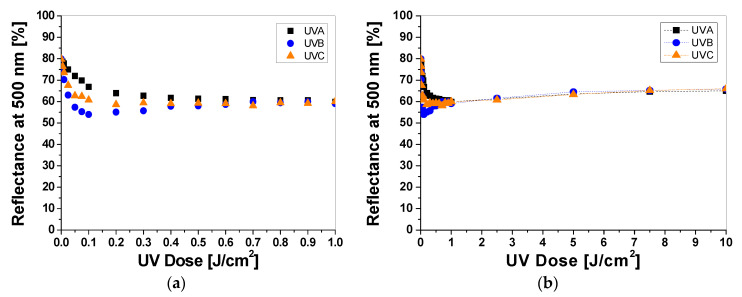
The reflectance of light at 500 nm vs. absorbed UV dose of printed cotton samples after UV irradiation (doses of UVA, UVB, and UVC in the range up to 1 J/cm^2^; samples were printed with TTC-containing paste and dried for 60 min at 30 °C) (**a**). Changes in light reflectance at 500 nm of samples irradiated with UV light doses up to 10 J/cm^2^ (the increase in light reflectance is due to the color bleaching) (**b**).

The intensity of the red color reaches a maximum after UVA, UVB, and UVC irradiation at 0.5 J/cm^2^, 0.1 J/cm^2^, and 0.2 J/cm^2^, respectively (Figure 4a). Thus, the difference in the light reflectance between the samples irradiated with UVA, UVB, and UVC at a dose of 0.1 J/cm^2^ equaled approx. 19% and 9%, respectively. In the range of 0.5 to 1.0 J/cm^2^, the samples showed no significant changes in reflectance, but above this dose, the samples changed their color to a stronger orange (Figure 4b). In the range of 0.5–1.0 J/cm^2^ the samples showed no significant changes in reflectance, but above this dose the change in reflectance of the sample irradiated with all UV subranges was very similar and amounted to approx. 6% at a dose of 5 J/cm^2^. To describe the color differences of the individual samples in detail, the CIE Lab values for arbitrarily chosen TTC-printed samples irradiated up to 10 J/cm^2^ were compared and presented in Table 2. Comparing the CIE Lab with TTC-printed samples irradiated with higher doses of UVB and UVC, a change in red color to more orange is clearly visible.

**Figure 5:**
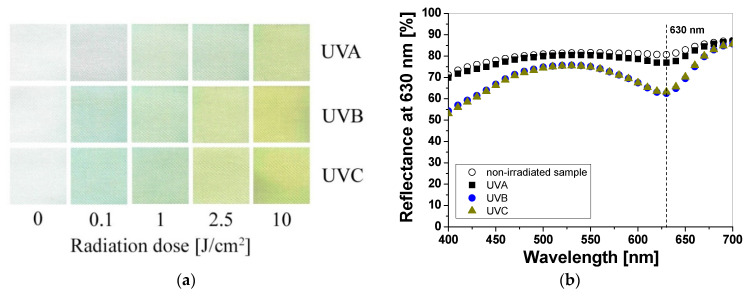
Photograph of cotton samples printed with LMG-containing printing paste and irradiated with UV light (UVA 369 nm; UVB 306 nm; and UVC 253.7 nm): 0, 0.1, 1, 2.5, and 10 J/cm^2^ (**a**) and reflectance spectra of the printed samples that were non-irradiated and irradiated with 1 J/cm^2^ of UVA, UVB, and UVC radiation (**b**).

**Figure 6:**
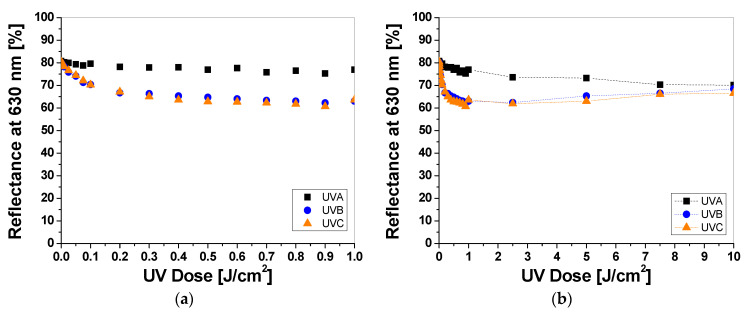
The reflectance of light at 630 nm vs. absorbed UV dose of the printed cotton samples after UV irradiation (doses of UVA, UVB, and UVC in the range up to 1 J/cm^2^; the samples were printed with LMG-containing paste and dried for 60 min at 30 °C) (**a**). Changes in light reflectance at 630 nm of samples irradiated with doses up to 10 J/cm^2^ of UV light (the increase in light reflectance is due to the color bleaching) (**b**).

UV-irradiated samples printed with LMG-containing printing paste change color from colorless to green (Figure 5a). As well as for LCV and TTC samples, the intensity of the color depends on the type of UV radiation and the absorbed radiation dose. The reflectance of the light decreases with the absorbed dose in the wavelength range 525–675 nm, with a maximum at 630 nm (Figure 5b). It can be seen, as for the printed samples with LCV, that the color change is more visible for the samples irradiated with UVB and UVC radiation. Similarly, the sensitivity of the dosimeter for UVA is the lowest of all the UV subranges (Figure 6a). For instance, the difference in light reflectance between the samples irradiated with UVA and UVB with a dose of 0.1 J/cm^2^ equaled approx. 11%, while the difference between the samples irradiated with UVB and UVC equaled approx. 0.2%. Moreover, as in the case of samples printed with LCV-containing pastes, no bleaching effect can be seen for the samples irradiated with UVA for the examined dose range (Figure 6b). The intensity of the green color reaches a plateau above 2.5 J/cm^2^. It should be noted that it is known that LMG solutions, under photochemical or chemical oxidation and reduction, can be bleached. Bleaching under oxidation in aerobic conditions can induce the demethylation of N–Me dyes or the cleavage of the central Ar–C bond, thus leading to amino-substituted benzophenones [20,21]. To describe the color differences of individual samples in detail, the CIE Lab values for chosen LCV-printed samples irradiated with up to 10 J/cm^2^ were compared and presented in Table 3. Comparing the CIE Lab with LMG-printed samples irradiated with higher doses of UVB and UVC, a change from a green color to a more yellow color is clearly visible.

Several parameters may influence the color of a printed pattern and its resistance to mechanical properties, such as the type and concentration of the binding agent and the temperature and time of drying the printing paste. In this work, a binding agent commonly used in the color printing of textiles was chosen. Its concentration in a printing paste was as recommended by the manufacturer. To determine the color changes of pure printing paste after UV irradiation in the range of up to 10 J/cm^2^ of UV radiation, samples were also printed without the addition of radiation-sensitive compounds. Based on the obtained results, it was found that no significant changes were observed in the range of UV radiation doses used in the study. For example, the color change between the samples irradiated with 0 and 10 J/cm^2^ was 0.30%, 0.31%, and 0.28% for UVA, UVB, and UVC, respectively.

### 3.2. Two-Color Printing

**Figure 7:**
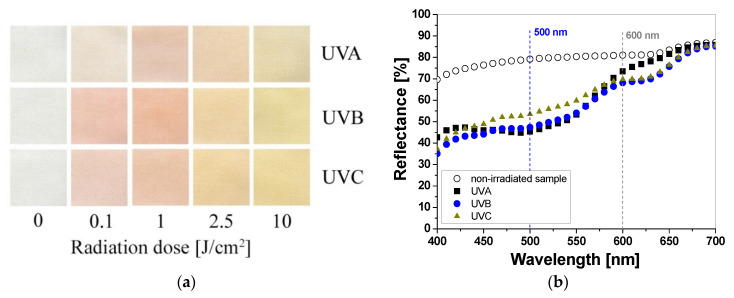
Photograph of cotton samples printed with LMG/TTC-containing printing paste and irradiated with UV light (UVA 369 nm; UVB 306 nm; and UVC 253.7 nm): 0, 0.1, 1, 2.5, and 10 J/cm^2^ (**a**) and reflectance spectra of the printed samples that were non-irradiated and irradiated with 1 J/cm^2^ of UVA, UVB, and UVC radiation (**b**).

**Figure 8:**
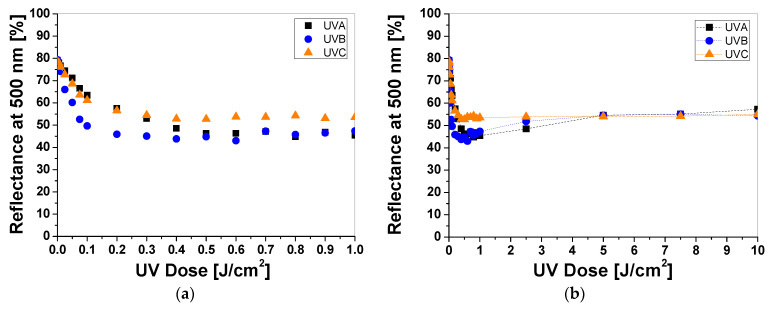
The reflectance of light at 500 nm vs. absorbed UV dose of the printed cotton samples after UV irradiation (doses of UVA, UVB, and UVC in the range up to 1 J/cm^2^; the samples were printed with LMG/TTC-containing paste and dried for 60 min at 30 °C) (**a**). Changes in light reflectance at 500 nm of samples irradiated with doses up to 10 J/cm^2^ of UV light (the increase in the light reflectance is due to the color bleaching) (**b**).

In the case of two-color prints, individual layers were applied one on top of the other to obtain a broader color palette. From the preliminary tests, no significant changes in the color were confirmed depending on the sequence of applied pastes containing radiation-sensitive compounds. For instance, the difference in color for the printed TTC/LMG and LMG/TTC cotton samples irradiated with 0.1 J/cm^2^ of all UV radiation subranges was below 0.2%, which can be considered a measurement error resulting from the accuracy of the reflectance spectrophotometer used in the research. The cotton samples were still white after printing, and the difference in color and sensitivity of dosimeters occurred after irradiation. The printed samples of LMG/TTC, LCV/TTC, and LCV/LMG changed their color from orange to yellow, from purple to brown, and from blue to gray, respectively. Samples printed with a paste containing TTC and LMG irradiated with UVA, UVB, and UVC changed color from colorless to orange/yellow. As for samples printed with one layer of TTC or LMG paste, the intensity of the color depended on the type of UV radiation (subrange) and the absorbed radiation dose (Figure 7a). For the two color blends, the wavelength of light at which the highest changes in the reflectance spectrum were observed had to be determined again. For the samples printed with LMG/TTC, the reflectance of light decreased with absorbed dose in the wavelength range of 450–660 nm, with a maximum of 500 nm (Figure 7b). The color intensity is related to the absorbed UV dose, and the higher the dose, the more intense the orange color was (Figure 8). It can be seen that the color change was more visible for the samples irradiated with UVB radiation. The sensitivity of the dosimeter to UVB was the highest of all UV subranges (Figure 8a). For instance, the difference in the light reflectance between the samples irradiated with UVA and UVC with the dose of 0.1 J/cm^2^ equaled approx. 4%, while the difference between UVB- and UVA-irradiated samples equaled approx. 22%. The intensity of the orange color reached a plateau above 0.5 J/cm^2^, as can be deduced from Figure 8a. No significant changes in the reflectance were observed for the dose range of 0.5–1 J/cm^2^, which indicates that saturation of the color occurred. However, irradiation over 1 J/cm^2^ of UVA and UVB radiation increased the reflectance of the samples (Figure 8b), similar to samples printed with one layer of TTC-containing printing paste. In the case of LMG/TTC prints, there was an additional quenching of the colors produced after irradiation.

According to the color theory, opposite colors, such as red and green, mixed in the same proportions create a gray color [10]. However, in this case, color mixing does not physically occur before the printing process. Immediately after printing, the non-irradiated cotton samples were white in color, whereas after irradiation in both layers of printing superimposed on each other, the appearing colors were compensated in the field of observation. Thus, the dependence of light reflectance as a function of wavelength is similar to the samples printed with these colors separately, but the nature of color changes after UV irradiation combines the properties of TTC and LMG. To describe the color differences of individual samples in detail, the CIE Lab values for chosen LMG/TTC-printed samples irradiated with up to 10 J/cm^2^ were compared and presented in Table 4. Comparing the CIE Lab with LMG/TTC-printed samples irradiated with higher doses of UVB and UVC, a change from an orange color to a more yellow color is clearly visible. The color fading effect described above is also noticeable.

**Figure 9:**
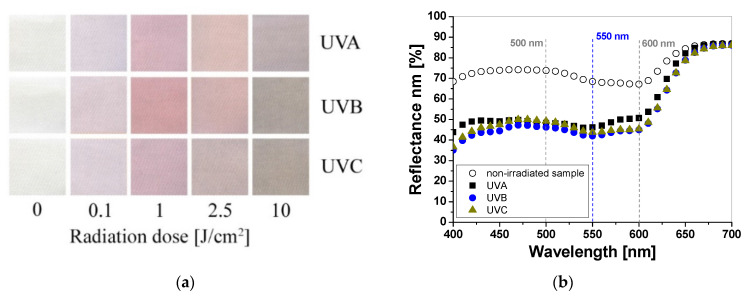
Photograph of cotton samples printed with LCV/TTC-containing printing paste and irradiated with UV light (UVA 369 nm; UVB 306 nm; and UVC 253.7 nm): 0, 0.1, 1, 2.5, and 10 J/cm^2^ (**a**) and reflectance spectra of the printed samples that were non-irradiated and irradiated with 1 J/cm^2^ of UVA, UVB, and UVC radiation (**b**).

**Figure 10:**
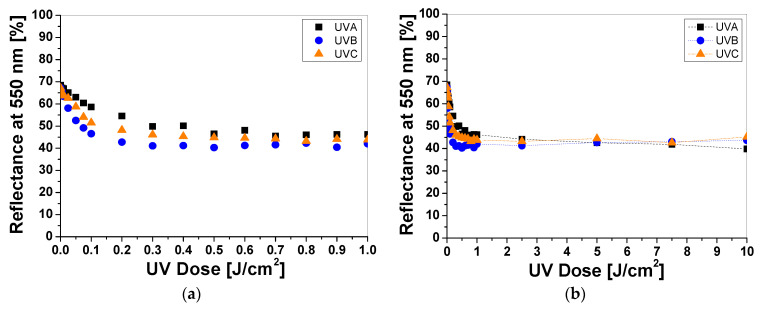
The reflectance of light at 550 nm vs. absorbed UV dose of the printed cotton samples after UV irradiation (doses of UVA, UVB, and UVC in the range up to 1 J/cm^2^; the samples were printed with LCV/TTC-containing paste and dried for 60 min at 30 °C) (**a**). Changes in light reflectance at 550 nm of samples irradiated with doses up to 10 J/cm^2^ of UV light (the increase in the light reflectance is due to the color bleaching) (**b**).

In the case of LCV/TTC-printed samples, no color compensation effect was observed after UV irradiation. Samples change color after irradiation from white to purple, and above a dose of 5 J/cm^2^, they become brown (Figure 9a). The reflectance of light decreases with absorbed dose in the wavelength range of 450–660 nm, with a maximum of 550 nm (Figure 9b). The color change and the sensitivity are similar in all three subranges of UV radiation. However, it is the most visible for UVB radiation (Figure 10a). For instance, the difference in the light reflectance for samples irradiated with UVA and UVC at a dose of 0.1 J/cm^2^ equaled approx. 12%, while the difference between UVA- and UVB-irradiated samples equaled approx. 21%. The intensity of the orange color reaches a plateau above 0.5 J/cm^2^ for samples irradiated with UVB and UVC (Figure 10b).

No significant changes in the reflectance were observed for the dose range of 0.5–1 J/cm^2^, and above a dose of 8 J/cm^2^, discoloration of the samples is visible. However, samples irradiated with UVA change their intensity up to 10 J/cm^2^. The difference in light reflectance for doses 0.5 and 10 J/cm^2^ equaled approx. 14%. In addition, changes in light reflectance as a function of wavelength have a slightly different character than in the case of the LMG/TTC combination. Mixing non-complementary colors allowed us to obtain a dosimeter that does not bleach above a dose of 1 J/cm^2^. To describe the color differences of individual samples in detail, the CIE Lab values for chosen LCV/TTC-printed samples irradiated up to 10 J/cm^2^ were compared and are presented in Table 5. Comparing the CIE Lab with LCV/TTC-printed samples irradiated with higher doses of UVA, UVB and UVC, a change from a purple color to a more brown/gray color is clearly visible.

**Figure 11:**
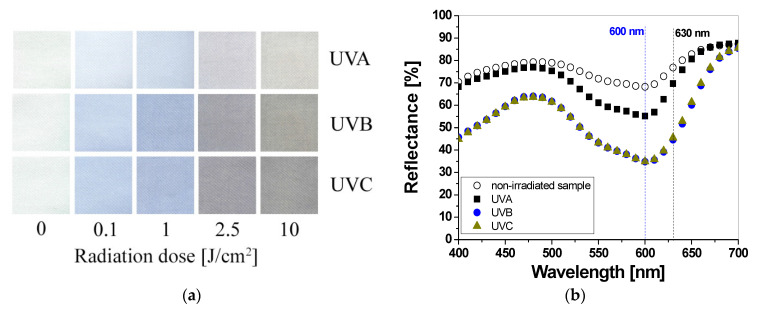
Photograph of cotton samples printed with LCV/LMG-containing printing paste and irradiated with UV light (UVA 369 nm; UVB 306 nm; and UVC 253.7 nm): 0, 0.1, 1, 2.5, and 10 J/cm^2^ (**a**) and reflectance spectra of the printed samples that were non-irradiated and irradiated with 1 J/cm^2^ of UVA, UVB, and UVC radiation (**b**).

**Figure 12:**
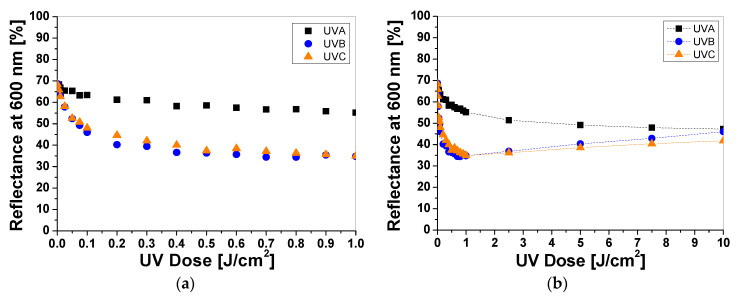
The reflectance of light at 600 nm vs. absorbed UV dose of the printed cotton samples after UV irradiation (doses of UVA, UVB, and UVC in the range up to 1 J/cm^2^; the samples were printed with LCV/LMG-containing pastes and dried for 60 min at 30 °C) (**a**). Changes in light reflectance at 600 nm of samples irradiated with doses up to 10 J/cm^2^ of UV light (the increase in the light reflectance is due to the color bleaching) (**b**).

Samples printed with LCV/LMG pastes changed color upon UV irradiation from white to blue and gray above a dose of 2 J/cm^2^ (Figure 11a). The reflectance of light decreased with absorbed dose in the wavelength range of 450–660 nm, with a maximum of 600 nm (Figure 11b). Samples irradiated with UVB and UVC radiation showed similar changes in the reflectance of light, which are presented in Figure 12a. Above the dose of 1 J/cm^2^, an increase in the reflectance value was visible, which resulted from the bleaching of the color of the samples (Figure 12b). Samples printed with LCV/LMG are less sensitive to UVA radiation. For instance, the difference in the light reflectance between the samples irradiated with UVB and UVC at a dose of 0.1 J/cm^2^ equaled approx. 4%, while the difference between the UVA- and UVB-irradiated samples equaled approx. 27%. For the samples irradiated with UVB and UVC, the intensity of the blue color reached a plateau above 1 J/cm^2^ and for the higher doses, an increase in reflectance and a change in color to gray were observed (Figure 12), whereas samples irradiated with UVA did not show the bleaching effect and registered dose changes in the range of up to 10 J/cm^2^. The nature of color changes after UV irradiation combined the properties of LCV and LMG. To describe the color differences of individual samples in detail, the CIE Lab values for chosen LCV/LMG-printed samples irradiated up to 10 J/cm^2^ were compared and are presented in Table 6. Comparing the CIE Lab with LCV/LMG-printed samples irradiated with higher doses of UVA, UVB, and UVC, a change in color from blue to gray is clearly visible.

### 3.3. Preparation of Printing Paste

**Figure 13:**
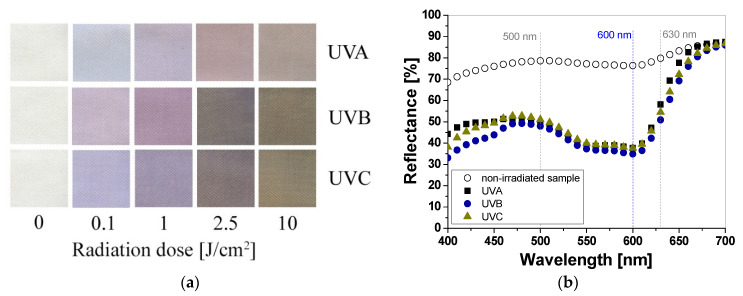
Photograph of cotton samples printed with LCV/LMG/TTC-containing printing pastes and irradiated with UV light (UVA 369 nm; UVB 306 nm; and UVC 253.7 nm): 0, 0.1, 1, 2.5, and 10 J/cm^2^ (**a**) and reflectance spectra of the printed samples that were non-irradiated and irradiated with 1 J/cm^2^ of UVA, UVB, and UVC radiation (**b**).

The color change of the samples printed with LCV/LMG/TTC combines the nature of all the used color precursors. White-printed samples change their color after irradiation to blue, purple, gray, and brown (Figure 13a) depending on the type of UV radiation. The reflectance of light decreases with absorbed dose in the wavelength range of 450–660 nm with a maximum of 600 nm (Figure 13b).

**Figure 14:**
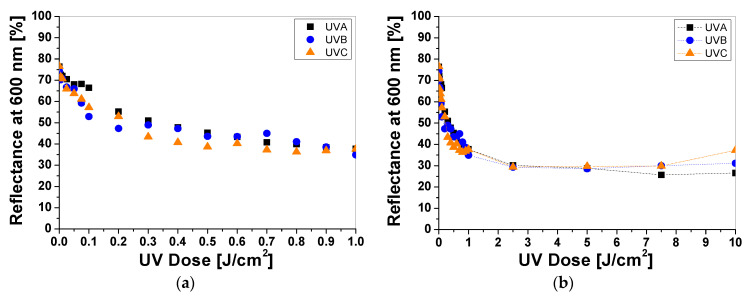
The reflectance of light at 600 nm vs. absorbed UV dose of the printed cotton samples after UV irradiation (doses of UVA, UVB, and UVC in the range up to 1 J/cm^2^; the samples were printed with LCV/LMG/TTC-containing pastes and dried for 60 min at 30 °C) (**a**). Changes in light reflectance at 600 nm of samples irradiated with doses up to 10 J/cm^2^ of UV light (the increase in the light reflectance is due to the color bleaching) (**b**).

The combination of color precursors with different characteristics allowed for the development of dosimeters with a wider measuring range for subranges of UV radiation. Thus, samples irradiated with UV radiation show similar changes in the reflectance of light up to 5 J/cm^2^, which is presented in Figure 14. Above the dose of 7 J/cm^2^, an increase in the reflectance value is visible for samples irradiated with UVC, which results from the bleaching of the color of the samples (Figure 14b). Samples printed with LCV/LMG/TTC are less sensitive to UVA radiation, but at the same time their measuring range is the widest. For instance, the difference in light reflectance between the samples irradiated with UVA and UVB at a dose of 0.1 J/cm^2^ equaled approx. 20%, while the difference between the UVA- and UVC-irradiated sample equaled approx. 14%. For the samples irradiated with UVB and UVC, the intensity of the blue color reaches a plateau above 2.5 J/cm^2^, and for the higher doses up to 7 J/cm^2^, an increase in reflectance and a change in color to brown are observed (Figure 14b), whereas samples irradiated with UVA do not show the bleaching effect and register dose changes in the range of up to 10 J/cm^2^. The nature of color changes after UV irradiation combines the properties of LCV, LMG, and TTC. To describe the color differences of individual samples in detail, the CIE Lab values for chosen printed samples irradiated up to 10 J/cm^2^ were compared and are presented in Table 7. Comparing the CIE Lab for LCV/LMG/TTC-printed samples irradiated with higher doses of UVA, UVB, and UVC, a change in color from purple to brown is clearly visible.

### 3.4. Stability and Basic Parameters of Printed Samples

**Figure 15:**
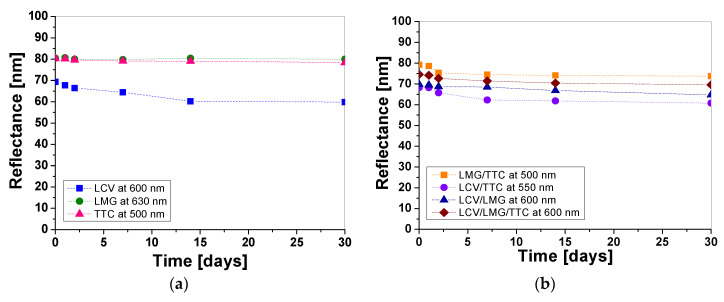
Evaluation of the stability of non-irradiated dosimeter samples over 30 days printed with pastes containing one (**a**), two, and three (**b**) color precursors.

**Figure 16:**
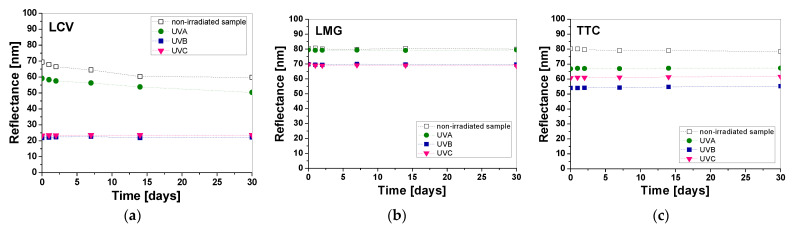
Evaluation of the stability of irradiated dosimeter samples with 0.1 J/cm^2^ of UVA, UVB, and UVC radiation over 30 days, printed with pastes containing one color precursor: LCV (**a**), LMG (**b**), and TTC (**c**).

**Figure 17:**
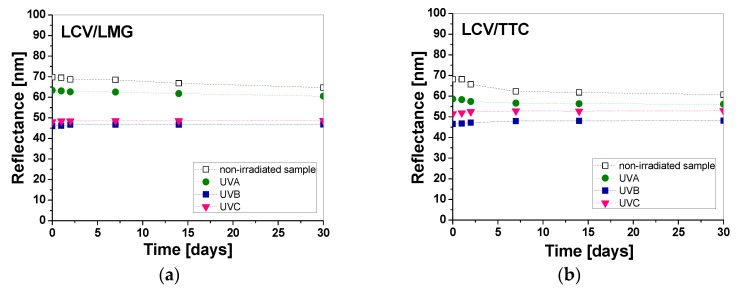

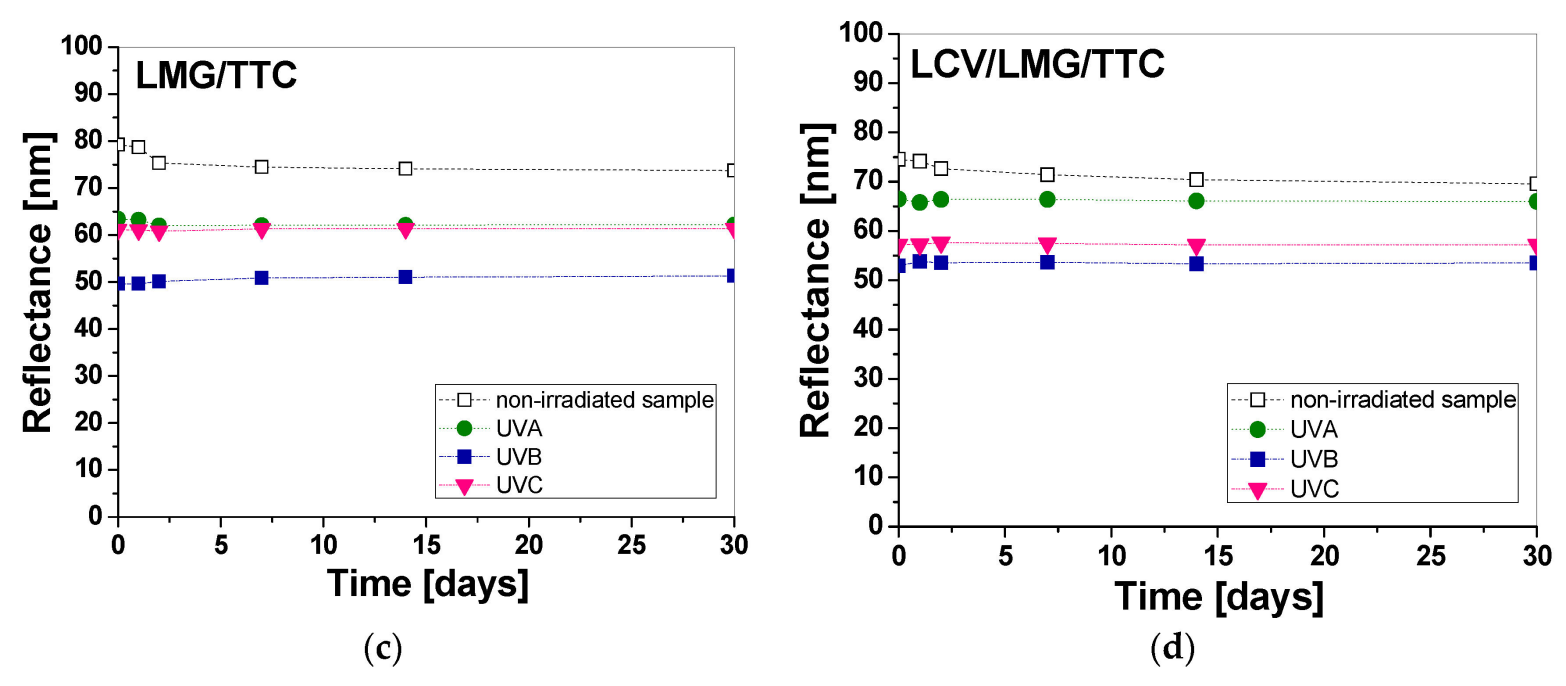
Evaluation of the stability of irradiated dosimeter samples with 0.1 J/cm^2^ of UVA, UVB, and UVC radiation over 30 days printed with pastes containing: LCV/LMG (**a**), LCV/TTC (**b**), LMG/TTC (**c**), and LCV/LMG/TTC (**d**).

It was noticed that some samples printed with different pastes containing color precursors may not be stable over time, despite being covered with an aluminum foil between the reflectance measurements. Therefore, the reflectance of the samples printed with different layers, both non-irradiated and irradiated with UVA, UVB, and UVC (0.1 J/cm^2^), was measured over time of storage for 30 days (Figure 15, Figure 16 and Figure 17). In the case of one-color prints, the samples printed with LMG and TTC show good stability, while for LCV the change in light reflectance is visible within the first 15 days from the printing of the samples (Figure 15a). The color change of the non-irradiated samples is due to the spontaneous transformation of LCV. It should be mentioned that, in this study, we did not cover the optimization of the dosimeter stability, which might be achieved by adding an underprint layer, adjusting the pH, or adding UV retarders to lower the sensitivity of the color precursors used in printing pastes. However, these changes may affect the dose–response performance of the dosimeter.

The reflectance measurements showed that the color change of the non-irradiated samples within 30 days was equal to 13.7%, 2.3%, and 0.7% for the samples printed with pastes containing LCV, TTC, and LMG, respectively. Based on the obtained data, it was found that the dosimeter printed with LMG seems to be the most stable over time. Similar stability measurements were performed for two- and three-color-printed samples. According to the results, the samples printed with LMG/TTC had the best stability within 30 days (Figure 15b). The reflectance measurements showed that the color change of the non-irradiated samples printed with two and three colors was equal to 10.9%, 7.1%, 6.9%, and 6.7% for the samples printed with pastes containing LCV/TTC, LCV/LMG, LMG/TTC, and LCV/LMG/TTC, respectively. To better characterize the in-time color changes of samples immediately after printing and after 30 days of storage, the CIE Lab color coordinates and the degree of whiteness were determined for all non-irradiated samples and are presented in Table 8 and Table 9.

The stability of the samples exposed to UV radiation after printing was similarly assessed. Printed samples were irradiated with a chosen dose (0.1 J/cm^2^ UVA, UVB, and UVC) and characterized by reflectance measurements for 0–30 days of storage (Figure 16 and Figure 17). In the case of samples printed with pastes containing TTC and LMG, the samples showed good stability over 30 days of evaluation. The color change of the samples after UVA irradiation with all subranges does not exceed 1.4% and 1.5% for LMG and TTC, respectively, while for LCV-printed samples, the color change is 14.9%, 2.1%, and 1.4% for UVA, UVB, and UVC, respectively. On the other hand, samples printed with two and three color precursors after UVB and UVC irradiation are stable regardless of the combination of printing pastes used. Color change within 30 days does not exceed 1.8%, 3.4%, 3.4%, and 1.2% for LCV/LMG, LCV/TTC, LMG/TTC, and LCV/LMG/TTC, respectively. However, there are larger differences for UVA irradiation, where the color changes are 4.6%, 4.3%, 2.0%, and 1.7% for LCV/LMG, LCV/TTC, LMG/TTC, and LCV/LMG/TTC, respectively. Thus, the smallest changes in the color of samples after irradiation were observed for printed samples: LMG, TTC, and LCV/LMG/TTC.

**Figure 18:**
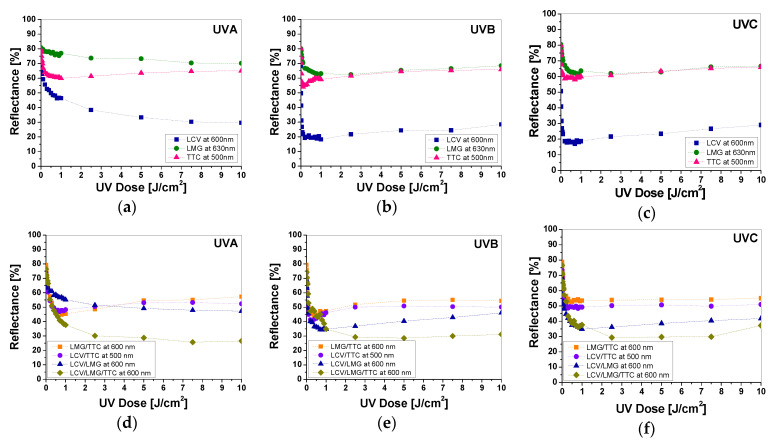
Evaluation of single- (**a**–**c**), two-, and three-color- (**d**–**f**) printed dosimeters in terms of selecting the optimal system for measuring UV radiation in the range 0–10 J/cm^2^.

The measurements of light reflectance were also used to determine the characteristic features of printed dosimeters with single, two, and three colors, such as threshold dose (R_0_), which is the minimum dose of radiation needed to cause a visible change of the light reflectance spectrum of the sample; dynamic dose response range, which is the response of the system to the dose until saturation (the plateau of the calibration curve), linear dose range, and dose sensitivity of the system, which is the slope of the linear regression. Moreover, for all samples, the linear measurement range was described by the linear equation A = a × D + A_0_, while the dynamic dose response range was described by the exponential equation A = A_1_ × exp(−D/t_1_) + A_2_ × exp(−D/t_2_) + A_0_, where D is dose and A corresponds to reflectance in both equations. All parameters are presented in Supplementary Tables S1–S3. In addition, Figure 18 presents the spectra of light reflectance as a function of UV radiation dose for samples printed with all color precursors to facilitate the assessment of characteristic parameters of the developed dosimeters. Comparing samples printed with a single color, it can be concluded that, after UVA irradiation, samples printed with LCV paste have the lowest threshold dose, the widest measuring range, and a higher sensitivity to the radiation dose than samples printed with LMG and TTC (Supplementary Table S1). Similar changes are observed for LMG-printed samples; however, the dosimeter’s sensitivity to the dose is lower by approx. 53% compared to LCV-printed samples. In the case of UVB radiation, regardless of the color precursor, the selection of the optimal dosimeter is complicated by the bleaching effect of the samples above the dose of 1 J/cm^2^. It seems that the LMG-printed dosimeter is optimal due to the lack of rapid changes in the reflectance value in the dose range up to 1 J/cm^2^. However, as in the case of UVA radiation, the dosimeter printed with LCV shows the highest sensitivity to UVB radiation. On the other hand, the TTC-printed dosimeter was found to be the most optimal for UVC radiation. Changes for samples printed with two and three color precursors are less diverse. Considering two-color prints, it was found that the LCV/LMG print is optimal for measuring all three subranges of UV radiation. These samples show a wide measuring range and sensitivity, although above 1 J/cm^2^ for UVB and UVC samples, they become bleached. However, the basic characteristics of the samples (Supplementary Table S2) prove that UV radiation doses in the range of 1–10 J/cm^2^ can also be registered. As expected, the dosimeter printed with three LCV/LMG/TTC precursors turned out to be the most sensitive to UV radiation (Supplementary Table S3). The nature of the changes after irradiation is similar to the LCV/LMG samples, but in the case of UVB and UVC, the influence of TTC is more visible, which reduces the bleaching effect of the samples above 2 J/cm^2^. In addition, for LCV/LMG/TTC samples irradiated with UVB in the range of 0.5–1 J/cm^2^, a scatter of measurement points was observed. This effect may be due to the red and green color compensation created by the TTC and LCV, respectively.

### 3.5. Application of Developed UV Dosimeters

**Figure 19:**
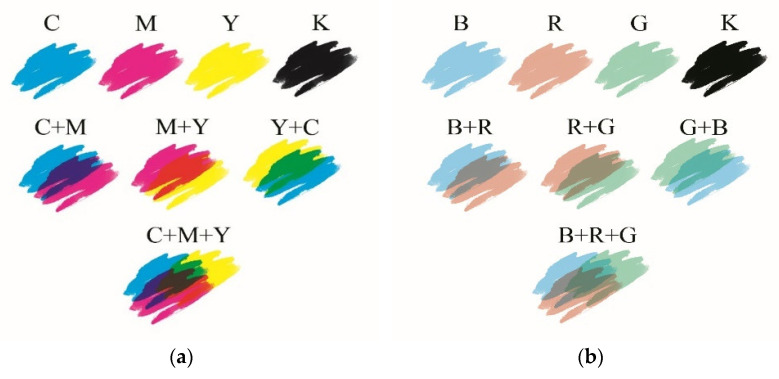
Comparison of colors based on the process colors of the standard CMYK system (cyan, magenta, yellow, and black) (**a**), and a modified system based on BRGK: blue (B from LCV), red (R from TTC), green (G from LMG), and black (not sensitive to UV radiation) (**b**).

Color formation via the additive and subtractive mixing of primary or process colors in appropriate proportions is commonly used for textile dyeing and printing. As a result of additively mixing the three primary colors, also known as the chromatic triplet, red, yellow, and blue, a wide range of colors can be achieved. The gamut of reproducible colors for a trichromatic system is limited and smaller than the gamut of all the possible colors that the average person can see. Additionally, the gamut range may be larger or smaller depending on the primary colors used [10]. Using an additive mixing system, it is impossible to obtain deep blacks in textile printing processes. Applying the standard CMYK color system based on the use of cyan, magenta, yellow, and black colors solves this problem, but this method is not widely used in the production of textiles due to the high time consumption and complexity of preparation for printing. Contrary to paper or foil-printed products, the main problem is the reproduction of pattern raster points on textile materials. Textiles printed using the CMYK color system method seem underdeveloped due to the low resolution of the created patterns. In addition, the raster printing process reduces the amount of printing paste transferred to the textile substrate, and hence the range of shades, color vividness, and quality of tonal transitions are inferior to other textile printing techniques such as roller or digital printing. Moreover, textiles absorb and reflect light differently than paper due to the structure and type of fibers. The highly porous, textured surface of the textiles influences the different absorption levels of the printing paste. However, the CMYK printing method is used to a limited extent in handmade clothing production. Despite the lack of literature and research in this area, it was found that the modified method of printing with the process colors can be used in this work. The use of transparent printing paste allowed for the mixing of the process colors without the need to split the pattern into raster points. Figure 19 presents the simulation of color formation in the standard CMYK system and the modified version with the use of LCV, LMG, and TTC dyes (the BRGK color system).

**Figure 20:**
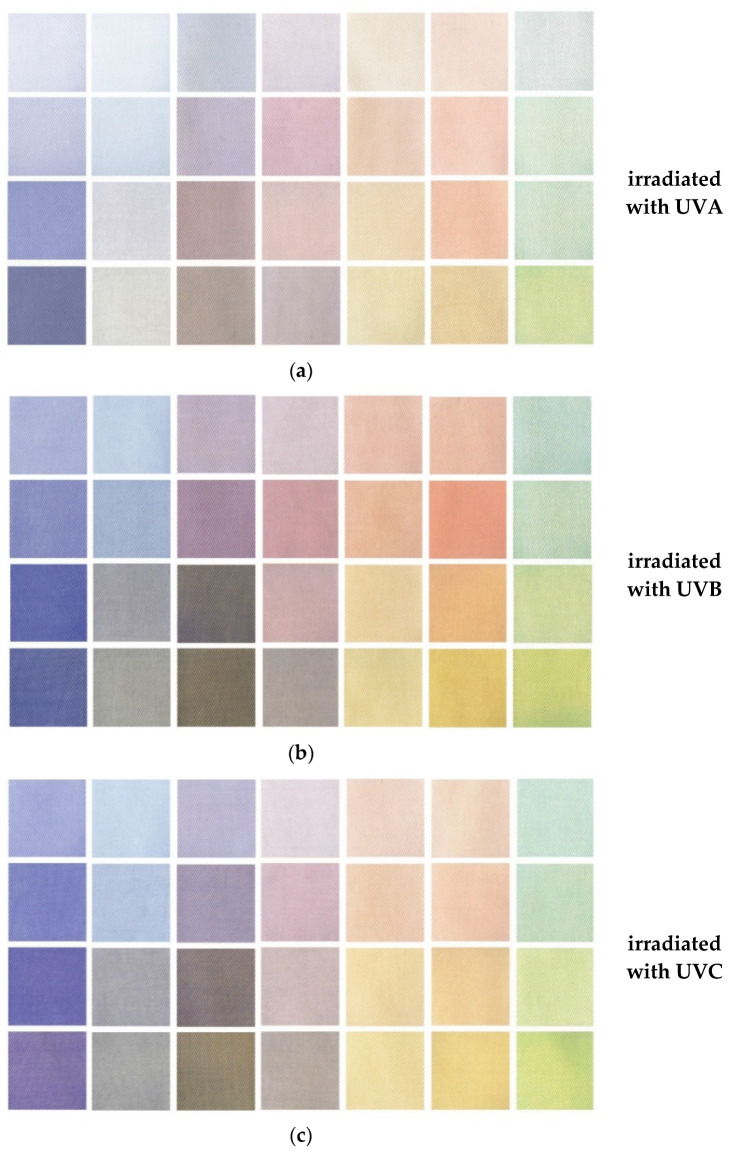
The color palette was produced as a result of UVA (**a**), UVB (**b**), and UVC (**c**) irradiation of cotton samples printed with single, two, and three colors.

The color derivatives obtained after UV irradiation are uniform and intense on the printed textile material. At this point, it is worth mentioning that in the presented work, color precursors were used at a concentration of 0.1%, which makes the color intensity lower in comparison to the process colors of the CMYK color system used in the screen and digital printing of textiles, where the concentration of the dye is usually about 5% [10]. The low concentration of the color precursors guaranteed that immediately after printing, the cotton samples would be white, without a visible color dominant. In addition, the low concentration of color precursors guaranteed a wide palette of color changes and in-time stability after UV irradiation, especially in the case of two- and three-color prints (Figure 20). Depending on the color precursors used for printing and their concentrations, the color effects may be different. Due to limitations in the production of tetrazolium salts and diacetylenes, these palettes do not contain pure primary colors. In the case of some radiation-sensitive precursors, in particular in the case of tetrazolium salts and diacetylenes, it is not possible to produce structures that would form primary colors as a result of UV irradiation.

Thus, the colors blue and green, and red and yellow cannot be obtained for tetrazolium salts and diacetylenes, respectively [22,23]. Nevertheless, based on the obtained data described in Section 3.1, Section 3.2, Section 3.3 and Section 3.4, it is possible to develop patterns that, depending on the UV dose or exposure time, will change their color and provide information about the absorbed dose. Thus, developed patterns can be used as personal dosimeters, markers of the authenticity and originality of textile products, decorative elements, and product freshness indicators, or design detailing elements and originality markers. It is also worth mentioning that simple markers are still being researched for various occupational groups exposed to accidental UV irradiation, e.g., for medical personnel, welders, beauty sector employees (especially nail stylists, solarium employees, and their clients), as well as astronauts. A properly designed marker, in the form of a user-friendly pattern, is also an eye-catcher because most of the occupations exposed to accidental UV irradiation use specific work wear.

The examination of the surface of samples printed with precursors sensitive to UV radiation was carried out using scanning electron microscopy (SEM). Due to the lack of noticeable differences between single-, two-, and three-color-printed samples before and after irradiation, these results are not presented in the manuscript. However, SEM images are shown for unprinted and three-color-printed cotton fabric both before and after UVB irradiation (Supplementary Figure S1). After printing, the filling of the structure of the textile product with printing paste is visible; however, there are no visible changes to the textile after UVB irradiation.

**Figure 21:**
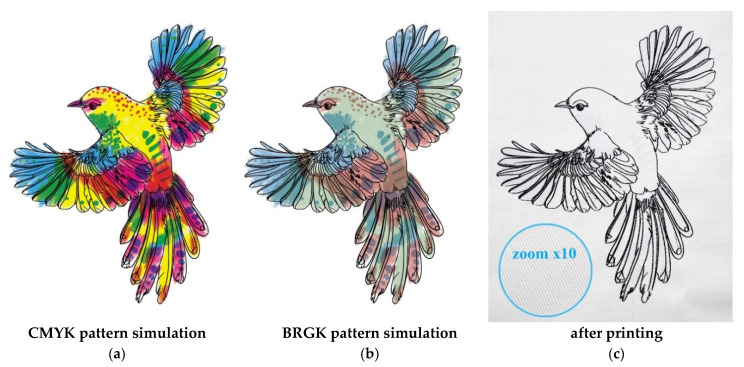
Visual effects after designing a graphic pattern for printing: (**a**) in the CMYK color system; (**b**) in the BRGK color system; and (**c**) after printing before irradiation (photography).

To determine the actual use of the developed dosimeters as elements of protective clothing, a pattern of a bird was designed and printed on the surface of the cotton fabric. After printing, a black outline of the developed pattern was visible on the surface of the fabric, while places printed with pastes containing LCV, LMG, and TTC remained white/transparent (Figure 21). The repeated fine lines visible on the fabric after printing (inset in Figure 21c) result from the structure of the woven fabric with a twill weave and are a characteristic feature of the printing substrate.

**Figure 22:**
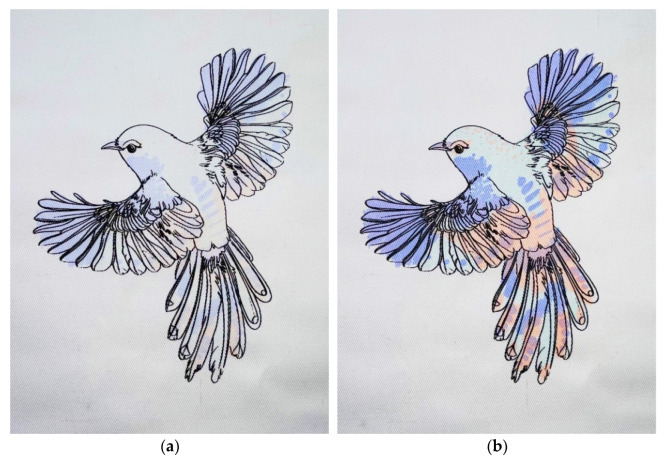

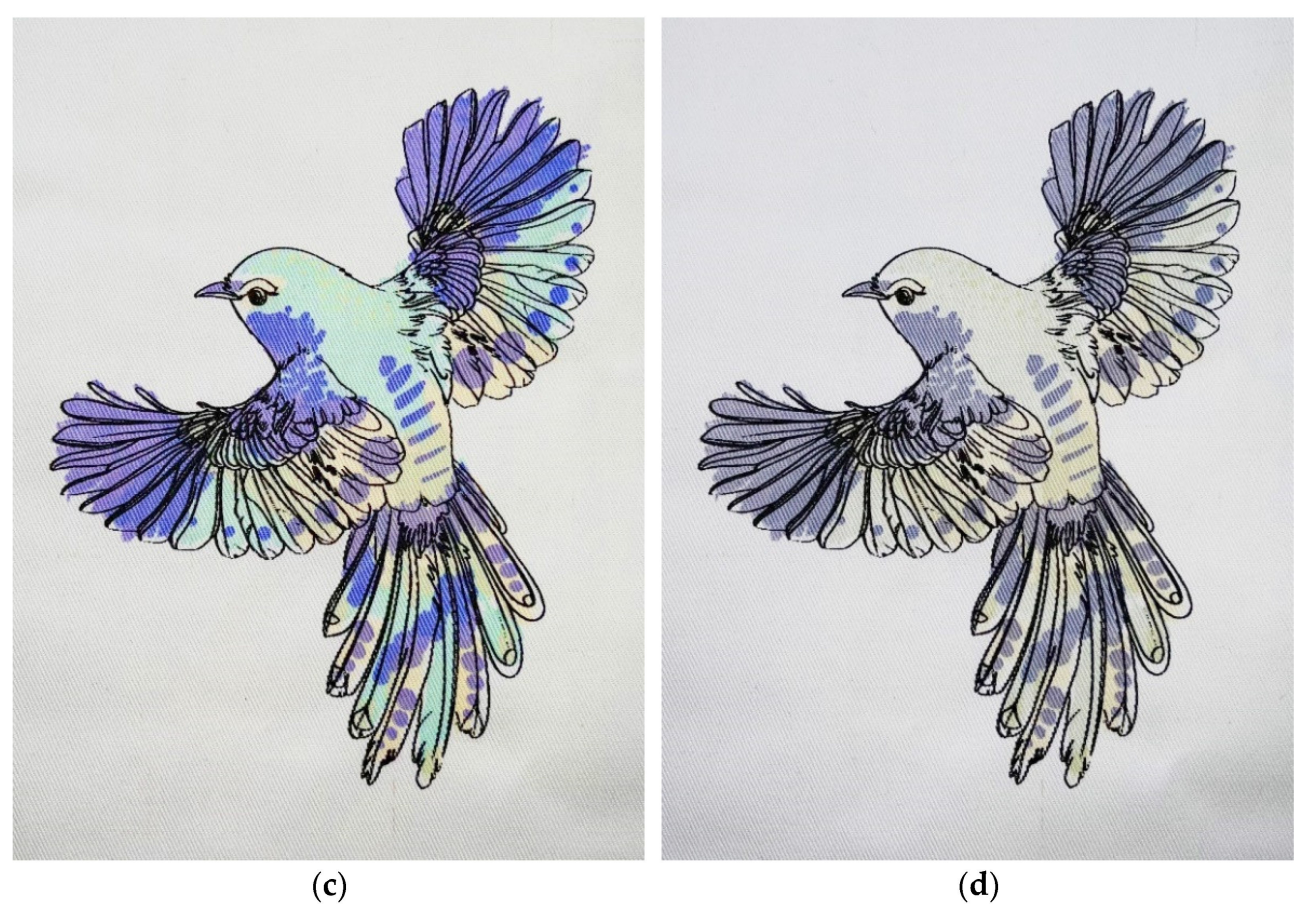
The printed three-colored pattern on cotton woven fabric after irradiation with UVB radiation: (**a**) 0.05 J/cm^2^; (**b**) 0.5 J/cm^2^; (**c**) 1 J/cm^2^; and (**d**) 10 J/cm^2^.

The developed three-colored prints were irradiated, similarly to the calibration samples, with UV radiation in the dose range of 0–10 J/cm^2^. As a result of irradiation, depending on the dose of radiation, colors were revealed (Figure 22) according to the mechanisms described in Section 3.1, Section 3.2, Section 3.3 and Section 3.4.

**Figure 23:**
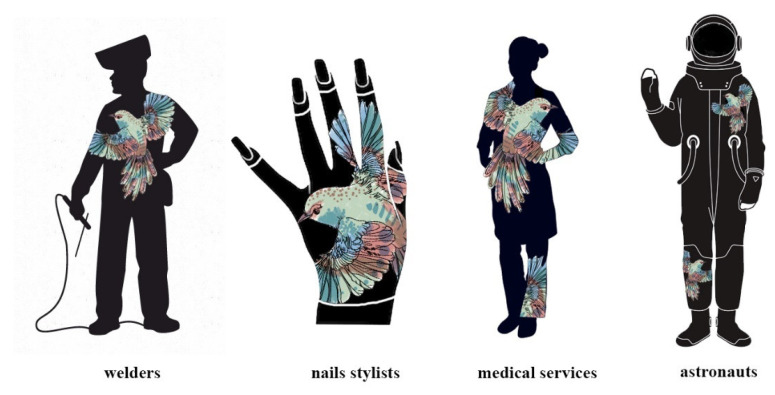
An example of patterns for printed markers on working clothes of various professional groups after UV irradiation. In the case of nail stylists and their clients, the marker is an element of protective gloves that shield against the effects of UV radiation emitted during the procedure.

Depending on the needs, the patterns can be varied in terms of size, arrangement on the printing substrate, or degree of complexity of the pattern. Figure 23 presents the potential possibilities of using such patterns printed on textile products. At the same time, in addition to UV radiation measurements, such markers can be used as elements for the protection of product originality and design detailing. It seems interesting to create a dosimeter that will combine the features of commercially produced markers with the possibility of obtaining a defined, colored pattern.

The developed dosimeters can also be used for radiation dose measurements as personal protection against the negative impact of UV radiation from natural and artificial sources of radiation on human skin. Research conducted in previous works proves that the color precursors used in the presented prints can be used for monitoring SED and MED doses, according to the International Commission on Non-Ionizing Radiation Protection, in cooperation with the World Health Organization [8,16]. If SED (10 mJ/cm^2^) and MED (25 mJ/cm^2^, Fitzpatrick type II skin) are taken into account, which denote 1 standard erythemal dose and 1 minimal erythemal dose, respectively, all the types of prints described above may be useful for such measurements due to their dose response range; however, because of their reported stability over time, they require further optimization.

## 4. Conclusions

This work introduces the new idea of reactive textile printing with a multi-color system using pastes containing radiation-sensitive compounds. It presents the development of UV radiation dosimeters via surface printing cotton fabrics with pastes containing radiochromic dyes, which change their color from colorless to blue, red, and green as a result of irradiation. It has been shown that the use of different color precursors and the number of printed layers on the fabric lead to different color effects. The BRGK printing method, based on printing pastes containing LCV, TTC, and LMG, can be successfully used as an alternative method to the multi-color CMYK printing on textiles and paper. The use of one-, two-, and three-color printing allowed us to obtain dosimeters for measuring UV radiation doses in the range of up to 10 J/cm^2^. It has also been shown that the use of a reflectance spectrophotometer allows us to obtain information about the color of dosimeters and the accurate characterization of the basic properties of such dosimeters, including: dose sensitivity, linear and dynamic dose response, and threshold dose. It was also shown that the developed dosimeters can be used for personal protection against UV radiation, marking, labeling, securing, decorative purposes, and design. Some of the described methods of marking textile and paper products have been reserved and are protected by a Polish patent P.419133.

The research results have not fully exhausted the subject and should be continued in terms of improving the stability over time and increasing the measurement range of the developed systems. In further work, the use of other leuco dyes, including other tetrazolium salts, or the use of other groups of radiation-sensitive dyes or polydiacetylenes, may be considered as alternatives to the current dyeing and printing methods. In addition, different printing pastes should be investigated, and changes in the components of the printing pastes should be considered. The change of color promoters or the addition of UV radiation retardants can lead to the production of systems with a wider measuring range and different dynamics of color development. Further examination may also include testing the washing fastness as an indicator of the durability of such prints and their daily use.

## Figures and Tables

**Table 1:** The influence of UV irradiation on color formation on cotton samples screen-printed with paste containing LCV.

UV Type	0 J/cm^2^			0.1 J/cm^2^			1 J/cm^2^			10 J/cm^2^		
	L	a	b	L	a	b	L	a	b	L	a	b
UVA	88.89	−0.76	−2.68	85.93	−0.97	−7.44	81.11	−0.55	−14.70	71.63	2.37	−26.25
UVB	88.68	−0.79	−3.06	66.13	4.90	−34.44	60.74	4.80	−31.61	65.95	0.23	−9.96
UVC	88.74	−0.80	−2.63	67.44	3.96	−32.58	61.16	4.36	−30.57	66.09	0.78	−10.13

**Table 2:** The influence of UV irradiation on color formation on cotton samples screen-printed with paste containing TTC.

UV Type	0 J/cm^2^			0.1 J/cm^2^			1 J/cm^2^			10 J/cm^2^		
	L	a	b	L	a	b	L	a	b	L	a	b
UVA	92.16	0.13	3.51	88.54	4.91	7.27	86.12	6.66	11.30	87.94	3.26	11.97
UVB	92.26	0.08	3.42	84.34	11.41	9.44	84.67	6.12	13.64	88.14	1.71	14.05
UVC	92.18	0.10	3.41	86.69	7.71	8.84	86.12	5.95	13.01	88.17	2.55	12.78

**Table 3:** The influence of UV irradiation on color formation on cotton samples screen-printed with paste containing LMG.

UV Type	0 J/cm^2^			0.1 J/cm^2^			1 J/cm^2^			10 J/cm^2^		
	L	a	b	L	a	b	L	a	b	L	a	b
UVA	92.20	−1.01	2.54	91.86	−1.37	3.02	91.48	−2.13	2.55	90.24	−4.30	1.91
UVB	92.06	−1.05	2.61	90.29	−4.50	2.53	88.12	−7.16	4.66	86.65	−3.60	11.03
UVC	92.09	−1.04	2.61	90.33	−4.55	2.30	88.05	−6.94	5.03	86.97	−5.25	9.62

**Table 4:** The influence of UV irradiation on the color formation on cotton samples screen-printed with LMG/TTC-containing paste.

UV Type	0 J/cm^2^			0.1 J/cm^2^			1 J/cm^2^			10 J/cm^2^		
	L	a	b	L	a	b	L	a	b	L	a	b
UVA	91.75	−0.20	2.98	87.17	6.24	6.66	80.60	14.42	11.97	84.28	3.00	14.93
UVB	91.73	−0.28	2.76	82.34	12.85	8.92	79.73	9.51	13.36	81.95	−0.89	19.49
UVC	91.54	−0.23	3.01	86.34	6.59	6.95	82.53	5.61	11.96	82.56	0.30	18.80

**Table 5:** The influence of UV irradiation on the color formation on cotton samples screen-printed with LCV/TTC-containing paste.

UV Type	0 J/cm^2^			0.1 J/cm^2^			1 J/cm^2^			10 J/cm^2^		
	L	a	b	L	a	b	L	a	b	L	a	b
UVA	86.33	1.18	−2.46	82.77	6.10	−0.14	76.12	8.95	0.94	72.42	2.07	−5.76
UVB	86.93	1.02	−3.24	76.80	13.69	1.89	72.51	7.50	1.10	73.60	−0.16	4.97
UVC	86.86	1.24	−2.78	79.32	9.53	−0.30	74.58	5.78	0.06	74.63	0.91	4.50

**Table 6:** The influence of UV irradiation on the color formation on cotton samples screen-printed with LCV/LMG-containing pastes.

UV Type	0 J/cm^2^			0.1 J/cm^2^			1 J/cm^2^			10 J/cm^2^		
	L	a	b	L	a	b	L	a	b	L	a	b
UVA	88.74	−1.30	−3.03	87.21	−1.61	−4.78	84.36	−1.83	−8.49	80.88	−2.43	−11.65
UVB	88.88	−1.35	−2.78	80.97	−4.16	−11.84	74.18	−6.57	−11.97	76.31	−2.51	3.31
UVC	88.74	−1.32	−2.78	81.85	−4.45	−10.41	74.19	−6.20	−11.74	74.76	−3.69	0.48

**Table 7:** The influence of UV irradiation on the color formation on cotton samples screen-printed with LCV/LMG/TTC-containing pastes.

UV Type	0 J/cm^2^			0.1 J/cm^2^			1 J/cm^2^			10 J/cm^2^		
	L	a	b	L	a	b	L	a	b	L	a	b
UVA	90.59	−0.63	1.33	85.31	3.33	0.67	71.98	5.19	−7.14	66.96	0.78	−13.67
UVB	90.08	−0.65	0.57	77.84	8.35	−2.14	70.34	0.79	−3.81	66.78	−2.73	3.88
UVC	90.69	−0.55	1.27	82.15	2.97	−3.20	72.34	1.80	−5.92	70.56	−1.61	4.98

**Table 8:** Color changes of non-irradiated samples within 30 days after printing with pastes containing color precursors.

Print Type	Day 0			Day 1			Day 7			Day 30		
	L	a	b	L	a	b	L	a	b	L	a	b
LCV	88.85	−0.81	−2.53	88.26	−0.82	−3.20	87.33	−0.91	−4.63	85.19	−0.82	−8.03
LMG	92.11	−1.05	2.68	91.98	−1.11	2.72	91.96	−1.20	2.81	91.41	−2.21	2.47
TTC	92.34	0.03	3.23	92.31	0.09	3.30	91.97	0.31	3.49	91.79	0.56	3.70
LCV LMG	89.06	−1.31	−1.87	88.97	−1.32	−1.91	88.68	−1.35	−2.38	87.53	−1.48	−4.00
LCV TTC	87.22	1.16	−2.45	87.21	1.34	−2.32	84.60	3.18	−3.77	83.91	3.42	−4.28
LMG TTC	91.71	−0.20	2.78	91.54	−0.06	2.88	90.44	1.41	3.58	90.06	1.28	3.49
LCV LMG TTC	90.21	−0.66	−2.28	90.04	−0.64	−2.47	89.16	−0.78	−3.59	88.31	−0.98	−5.43

**Table 9:** Whiteness changes of non-irradiated samples within 30 days after printing with pastes containing color precursors.

Whiteness	LCV	LMG	TTC	LCV/LMG	LCV/TTC	LMG/TTC	LCV/LMG/TTC
after preparation	88.6	68.8	66.8	86.4	84.4	66.0	76.83
after 30 days	87.0	67.7	63.1	84.5	83.7	59.5	79.58

## Data Availability

Data is available on request by contacting the corresponding authors.

## Supplementary Materials

The following supporting information can be downloaded at: https://www.mdpi.com/article/10.3390/ma16165622/s1, Table S1: Basic parameters of a single-color printed dosimeter irradiated with UVA, UVB, and UVC radiation, derived from the dose–responses; Table S2: Basic parameters of two-color printed dosimeter irradiated with UVA, UVB, and UVC radiation, derived from the dose–responses; Table S3: Basic parameters of three-color printed dosimeter irradiated with UVA, UVB, and UVC radiation, derived from the dose–responses; Figure S1: Scanning electron microscopy images of cotton textile samples unprinted, printed in three col-ors, before and after UVB irradiation.
