# Supplementary material for: Multi-Color Printed Textiles for Ultraviolet Radiation Measurements, Creative Designing, and Stimuli-Sensitive Garments
Source: Materials (Basel). 2023 Aug 14;16(16):5622. doi: 10.3390/ma16165622 (PMC10456908; doi:10.3390/ma16165622)
Supplement: Supplementary file 1 [file materials-16-05622-s001.zip › materials-2540625-supplementary (1)-done.pdf]

materials

MDPI

Supplementary material

Multi-Color Printed Textiles for Ultraviolet Radiation Measurements, Creative Designing, and Stimuli-Sensitive Garments

Elżbieta Sąsiadek-Andrzejczak* and Marek Kozicki*

Lodz University of Technology, Department of Mechanical Engineering, Informatics and Chemistry of Polymer Materials, Żeromskiego 116, 90-543 Lodz, Poland
* Correspondence: elzbieta.sasiadek@p.lodz.pl (E.S-A.); marek.kozicki@p.lodz.pl (M.K.); Tel.: +48-42-631-33-70 (E.S-A.); +48-42-631-33-77 (M.K.)

Abstract: This work concerns the new idea of textile printing with a multi-color system using pastes containing compounds sensitive to ultraviolet (UV) radiation. A screen printing method based on a modified CMYK color system was applied to a cotton woven fabric. Aqueous printing pastes were prepared from thickening and crosslinking agents and UV-sensitive compounds: leuco crystal violet (LCV), leuco malachite green (LMG), and 2,3,5-triphenyltetrazolium chloride (TTC) instead of the system's standard process colors: cyan, magenta, and yellow. Depending on the number of printed layers and the type of UV radiation (UVA, UVB, and UVC), the modified textile samples change color after irradiation from white to a wide range of colors (from blue, red, and green to purple, brown, and gray). Based on reflectance measurements, the characteristic parameters of the one-, two-, and three-color-printed samples in relation to absorbed dose were determined, e.g., dose sensitivity, linear and dynamic dose response, and threshold dose. This printing method is a new proposal for UV dosimeters and an alternative standard for textile printing. Furthermore, the developed method can be used for the securing, marking, and creative design of textiles and opens up new possibilities for such stimulus-sensitive reactive printing.

Keywords: textile printing, three-color printing, UV dosimeters, UV radiation monitoring, stimuli sensitive garments, creative designing of textiles

3. Results and discussion

3.4. Stability and Basic Parameters of Printed Samples

Based on the measurements of the light reflectance, the characteristics of single-, two, and three-color printed dosimeters were determined, such as the threshold dose (R_0), i.e., the minimum dose of radiation needed to cause a visible change in the reflection spectrum of the sample; the dynamic dose response range, which is the system's response to dose until saturation (the plateau of the calibration curve), the linear dose range, and the system's dose sensitivity, which is the slope of the linear regression. For all samples, the linear measurement range was described by the linear equation $A = a \times D + A_0$, while the dynamic dose response range was described by the exponential equation $A = A_1 \times \exp(-D/t_1) + A_2 \times \exp(-D/t_2) + A_0$, where D is dose and A corresponds to reflectance in both equations. All parameters are presented in Supplementary Tables S1-S3.

Supplementary Table S1. Basic parameters of a single-color printed dosimeter irradiated with UVA, UVB, and UVC radiation, derived from the dose–responses (Figures 2, 4, and 6)

Print type	UV type	Threshold Dose (J/cm²)	Linear Dose Range (J/cm²)	Dynamic Dose Range (J/cm²)	Linear Equation $A = a \times D + A_0$ where D is dose and A corresponds to reflectance			Exponential Equation $A = A_1 \times \exp(-D/t_1) + A_2 \times \exp(-D/t_2) + A_0$ where D is dose and A corresponds to reflectance					
					a (cm²/J)	A_0 (-)	R^2	A_1	t_1	A_2	t_2	A_0	R^2
TTC	UVA	0.01	0.01-0.1	0.005-10 (>2 bleaching)	-115.89±7.74	78.32±0.47	0.9823	6.12±8.76	1.36±3.66	16.22±1.47	0.08±0.01	57.13±10.16	0.9980
TTC	UVB	0.01	0.1-0.8	0.005-10 (>0.1 bleaching)	9.77±0.77	53.12±0.34	0.9629	24.12±1.48	0.02±0.01	1.48±1.71	-	54.34±0.43	0.9963
TTC	UVC	0.01	1-8	0.005-10 (>1 bleaching)	0.74±0.07	59.35±0.36	0.9674	7.65±10.11	0.06±0.05	13.20±10.03	0.02±0.01	59.12±0.21	0.9929
LCV	UVA	0.005	0.3-0.8	0.005-8	-12.65±1.07	56.46±0.62	0.9648	13.15±0.89	0.11±0.01	25.03±0.77	2.43±0.29	29.34±0.72	0.9960
LCV	UVB	0.005	0.1-0.9	0.005-10 (>1 bleaching)	-1.02±1.07	20.17±0.69	0.9600	19.97±2.87	0.04±0.01	28.58±2.94	0.11±0.01	19.53±0.25	0.9971
			1-10		0.98±0.17	18.29±1.08	0.8827						
LCV	UVC	0.005	1-10	0.005-10 (>1 bleaching)	1.02±0.07	18.78±0.48	0.9860	32.92±1.68	-	17.50±1.59	0.07±0.01	18.15±0.22	0.9982
LMG	UVA	0.01	0.1-0.9	0.005-10	-3.76±0.56	79.40±0.31	0.7832	3.27±1.22	0.36±0.24	9.85±4.27	8.10±8.21	67.05±5.18	0.9583
			1-10		-0.72±0.15	76.58±0.95	0.8362						
LMG	UVB	0.005	0.3-0.9	0.005-10 (>1 bleaching)	-6.50±0.3	68.02±0.20	0.9858	12.75±1.03	0.08±0.01	-12.35±47.87	-2.67±8.39	79.53±48.89	0.9977
			1-10		0.77±0.09	60.80±0.64	0.9567						
LMG	UVC	0.005	0.005-0.1	0.005-10 (>1 bleaching)	-99.50±5.85	79.73±0.30	0.9796	12.89±2.49	0.09±0.01	10.13±5.17	0.93±1.35	57.16±7.51	0.9979
			1-10		0.68±0.14	60.12±0.96	0.8826						

Supplementary Table S2. Basic parameters of two-color printed dosimeter irradiated with UVA, UVB, and UVC radiation, derived from the dose–responses (Figures 8, 10 and 12)

Print type	UV type	Threshold Dose (J/cm²)	Linear Dose Range (J/cm²)	Dynamic Dose Range (J/cm²)	Linear Equation $A = a \times D + A_0$ where D is dose and A corresponds to reflectance			Exponential Equation $A = A_1 \times \exp(-D/t_1) + A_2 \times \exp(-D/t_2) + A_0$ where D is dose and A corresponds to reflectance					
					a (cm²/J)	A_0 (-)	R^2	A_1	t_1	A_2	t_2	A_0	R^2
LMG TTC	UVA	0.01	0.01-0.1	0.005-10 (>1 bleaching)	-155.59±4.67	78.65±0.24	0.9946	16.63±0.01	0.18±0.01	16.63±0.01	0.18±0.02	45.48±0.48	0.9947
			1-10		1.29±0.25	45.44±1.55	0.8633						
	UVB	0.005	0.005-0.1	0.005-10 (>1 bleaching)	-329.93±27.89	76.66±1.17	0.9720	17.44±2.84	0.55±0.01	17.44±0.02	0.56±0.02	44.50±0.60	0.9952
	UVC	0.005	0.005-0.2	0.005-10 (>1 bleaching)	-200.36±7.93	78.46±0.30	0.9922	23.54±9.16	0.08±0.02	43.15±4.69	6.78±0.79	12.42±4.71	0.9971
			1-10		0.14±0.03	53.37±0.17	0.8574						
LCV TTC	UVA	0.005	0.005-0.1	0.005-10 (>1 bleaching)	-134.65±4.40	73.45±0.23	0.9936	16.96±1.92	0.12±0.35	10.31±1.89	0.22±1.39	46.58±2.91	0.9974
	UVB	0.005	0.3-1	0.005-10 (>0.3 bleaching)	9.65±0.38	37.60±0.22	0.9922	21.16±17.45	0.06±0.03	12.35±17.80	0.03±0.02	40.33±0.57	0.9992
	UVC	0.005	0.005-0.1	0.005-0.5 (>0.5 bleaching)	-229.58±17.23	72.08±0.66	0.9724	12.24±0.01	3.61±0.06	12.24±0.01	3.61±0.06	48.75±1.18	0.9918
LCV LMG	UVA	0.01	0.2-1	0.01-10	-7.43±0.82	62.34±0.53	0.9102	6.04±0.80	0.09±0.02	14.17±0.73	1.67±0.25	47.80±0.42	0.9914
	UVB	0.005	0.005-0.1	0.005-10 (>1 bleaching)	-193.95±18.60	64.04±1.04	0.9556	16.54±4.17	0.23±0.10	17.10±4.97	0.04±0.01	34.11±1.38	0.9959
			1-10		1.24±0.04	33.73±0.24	0.9960						
	UVC	0.005	0.2-1	0.005-10 (>1 bleaching)	-9.14±1.31	43.69±0.90	0.8714	18.27±0.99	0.34±0.05	15.75±1.26	0.03±0.01	34.36±0.76	0.9974
			1-10		0.86±0.03	34.04±0.16	0.9951						

Supplementary Table S3. Basic parameters of three-color printed dosimeter irradiated with UVA, UVB, and UVC radiation, derived from the dose–responses (Figure 14)

Print type	UV type	Threshold Dose (J/cm^2)	Linear Dose Range (J/cm^2)	Dynamic Dose Range (J/cm^2)	Linear Equation $A = a \times D + A_0$ where D is dose and A corresponds to reflectance			Exponential Equation $A = A_1 \times \exp(-D/t_1) + A_2 \times \exp(-D/t_2) + A_0$ where D is dose and A corresponds to reflectance					
					a (cm^2/J)	A_0 (-)	R^2	A_1	t_1	A_2	t_2	A_0	R^2
LCV LMG TTC	UVA	0.005	0.005-0.2	0.005-10	-84.29±7.21	73.18±0.66	0.9576	29.58±4.75	0.27±0.05	18.40±4.46	1.77±0.71	26.38±1.01	0.9946
			0.3-0.9		-24.98±1.95	58.13±0.62	0.9909						
	UVB	0.005	0.2-1	0.005-10 (>3 bleaching)	-15.28±2.39	52.51±1.56	0.8319	29.12±6.32	1.47±0.99	21.04±4.05	0.07±0.02	23.54±8.54	0.9708
	UVC	0.005	0.025-0.15	0.005-10 (>7 bleaching)	-143.99±17.78	71.41±0.77	0.9558	15.92±5.69	1.08±0.63	28.13±5.91	0.14±0.04	29.34±1.36	0.9867
			0.3-0.9		-12.66±2.69	46.40±1.55	0.8091						

After UVA irradiation, samples printed with LCV paste have the lowest threshold dose, the widest measuring range, and a higher sensitivity to the radiation dose than samples printed with LMG and TTC (Supplementary Table S1). Similar changes are observed for LMG printed samples, however, the dosimeter's sensitivity to the dose is lower by approx. 53% compared to LCV printed samples. In the case of UVB radiation, regardless of the color precursor, the selection of the optimal dosimeter is complicated by the bleaching effect of the samples above the dose of 1 J/cm^2. It seems that the LMG printed dosimeter is optimal due to the lack of rapid changes in the reflectance value in the dose range up to 1 J/cm^2. However, as in the case of UVA radiation, the dosimeter printed with LCV shows the highest sensitivity to UVB radiation. On the other hand, the TTC-printed dosimeter was found to be the most optimal for UVC radiation. Changes for samples printed with two- and three-color precursors are less diverse. Considering two-color prints, it was found that the LCV/LMG print is optimal for measuring all three sub-ranges of UV radiation. These samples show a wide measuring range and sensitivity, although above 1 J/cm^2 for UVB and UVC samples, they become bleached. However, the basic characteristics of the samples (Supplementary Table S2) prove that UV radiation doses in the range of 1–10 J/cm^2 can also be registered. As expected, the dosimeter printed with three LCV/LMG/TTC precursors turned out to be the most sensitive to UV radiation (Supplementary Table S3). The nature of the changes after irradiation is similar to the LCV/LMG samples, but in the case of UVB and UVC, the influence of TTC is more visible, which reduces the bleaching effect of the samples above 2 J/cm^2. In addition, for LCV/LMG/TTC samples irradiated with UVB in the range of 0.5–1 J/cm^2, a scatter of measurement points was observed. This effect may be due to the red and green color compensation created by the TTC and LCV, respectively.

3.5. Application of Developed UV Dosimeters

The examination of the surface of samples printed one-, two-, and thee-colors was carried out using scanning electron microscopy (SEM). Presented below SEM images of a

cotton fabric are shown for unprinted and three colour printed cotton fabric both before and after UVB irradiation (Supplementary Figure S1).

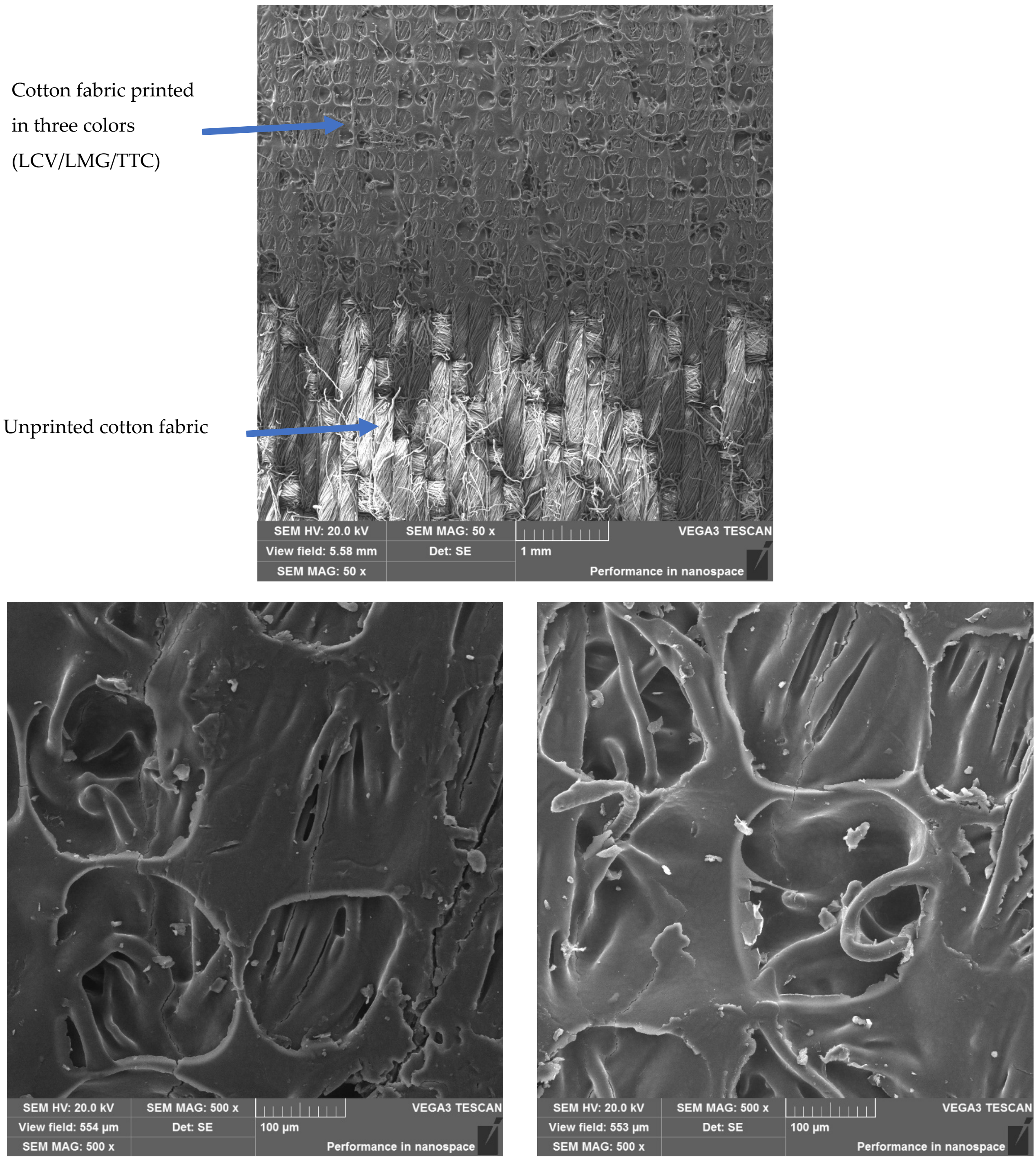

Cotton sample printed with LCV/LMG/TTC before irradiation

Cotton sample printed with LCV/LMG/TTC after UVB irradiation with a dose of 10 J/cm^2

Supplementary Figure S1. Scanning electron microscopy images of cotton textile samples unprinted, printed in three colors, before and after UVB irradiation.
